# Case Report: A rare case of male breast synovial sarcoma

**DOI:** 10.3389/fonc.2025.1469910

**Published:** 2025-05-26

**Authors:** Wang Yue, Ding Hua, Fu Yumei, Liu Mengyi, Zhang Jian, Song Dajiang, Liu Shu, Luo Ke

**Affiliations:** ^1^ Department of Breast Surgery, The Affiliated Hospital of Guizhou Medical University, Guiyang, Guizhou, China; ^2^ Department of Thoracic Surgery, The Affiliated Hospital of Guizhou Medical University, Guiyang, Guizhou, China; ^3^ Department of Breast Oncoplastic Surgery, Hunan Cancer Hospital, Changsha, China

**Keywords:** breast synovial sarcoma, chest wall reconstruction, breast reconstruction, vascular anastomosis, case report

## Abstract

Synovial sarcoma (SS) is a rare and aggressive malignancy that primarily affects young people and usually occurs in the extremities. Breast involvement is uncommon, and males with breast SS are even rarer. We present a rare case of a 17-year-old boy with a progressively growing mass found in his right breast, which was confirmed as SS through pathological diagnosis following surgery.

## 1 Introduction

Synovial sarcoma (SS) is a rare mesenchymal malignancy primarily affecting adolescents and young adults, commonly arising at the extremities (1, 2). Breast cancer in males is a rare condition, accounting for approximately 1% of all breast cancers and typically being estrogen receptor-positive (3). Breast SS in males is extremely rare. Currently, no such cases have been reported in the literature. This report describes the first known case of breast SS in a male.

## 2 Case presentation

**Figure 1:**
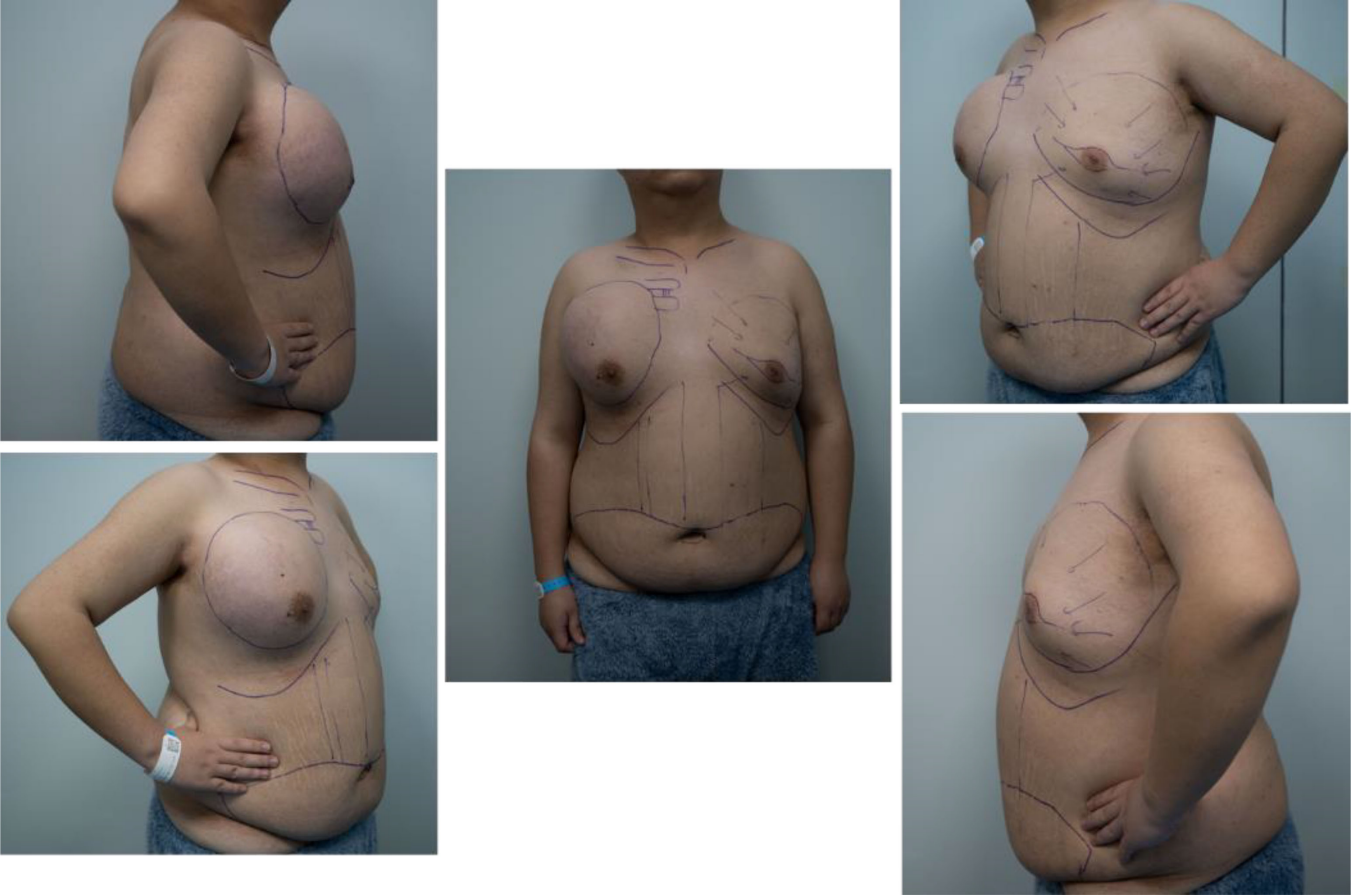
Preoperative pictures of the patient.

**Figure 2:**
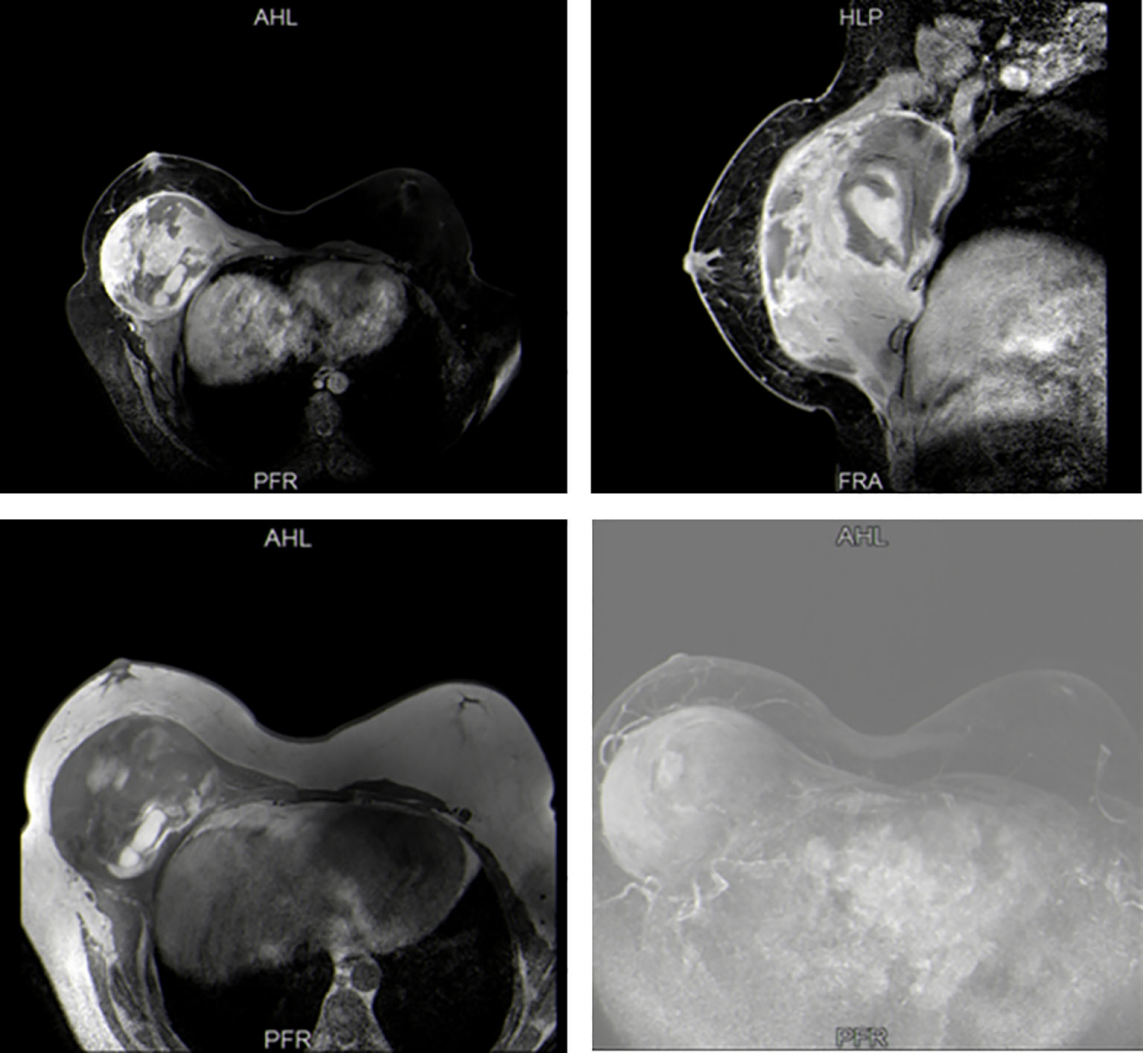
Preoperative Breast MRI.

A 17-year-old boy presented to our hospital with a 10-month history of a rapidly enlarging mass in right breast. The patient had no other obvious symptoms except for mild tenderness in the mass over the past 6 months. He did not seek medical attention promptly due to a lack of family history of breast cancer. On physical examination, a hard lump measuring 30 × 30 cm, with poor mobility and an indistinct boundary, occupying almost his entire right breast (**Figure 1**). Moreover, this boy had an enlarged left breast. He had no palpable lymphadenopathy, in the axilla, supraclavicular or cervical regions. Sex hormones tests showed increased levels of prolactin and progesterone. Breast ultrasound indicated gynecomastia in the left breast and a solid cystic mass in the right breast adjacent to the chest wall. Magnetic resonance imaging (MRI) confirmed a space-occupying lesion in the right breast and chest, accompanied by hemorrhage and necrosis (**Figure 2**). An ultrasound-guided biopsy puncture of the right breast was performed. Immunohistochemical staining results were as follows: Vim (+), Desmin (-), SMA (+), Calponin (+), Caldesmon (-), CK (+), EMA (+), CK7 (+), CK19 (+), SS18-SSX (+), TLE1 (+), CK5/6 (-), CKL (-), CKH (-), P63 (-), S100 (+),sox10 (-), CD57 (+), GFAP (-), ER (+), PR (-), CD34 (-), STAT6 (-), BCL-2 (+), CD99 (+), and Ki-67 (+). Furthermore, fluorescence *in situ* hybridization (FISH) confirmed SS18 gene translocation. Based on these findings, the patient was diagnosed with SS in the right breast and gynecomastia in the left breast.

## 3 Treatment

**Figure 3:**
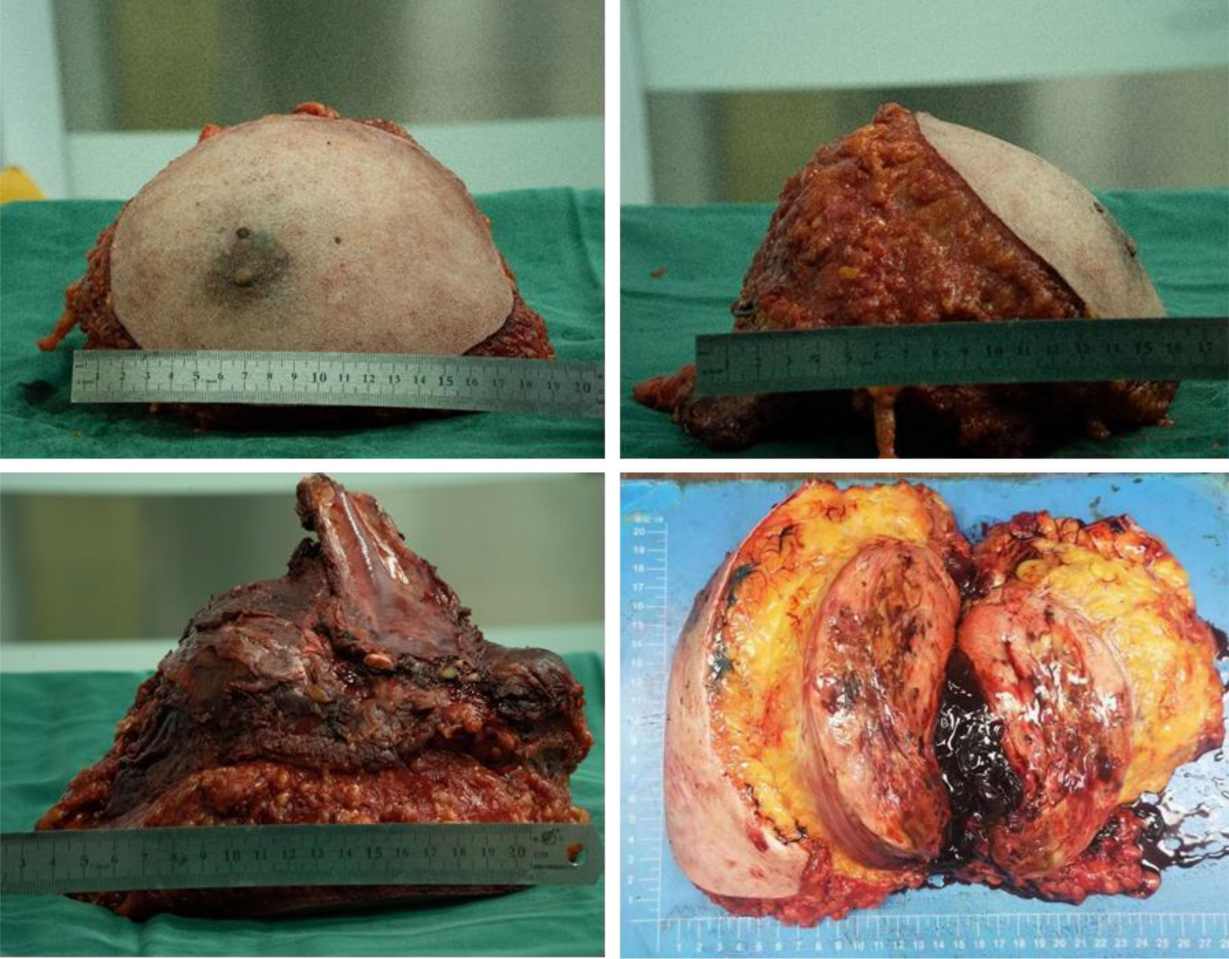
Extended radical mastectomy of right breast tumor.

**Figure 4:**
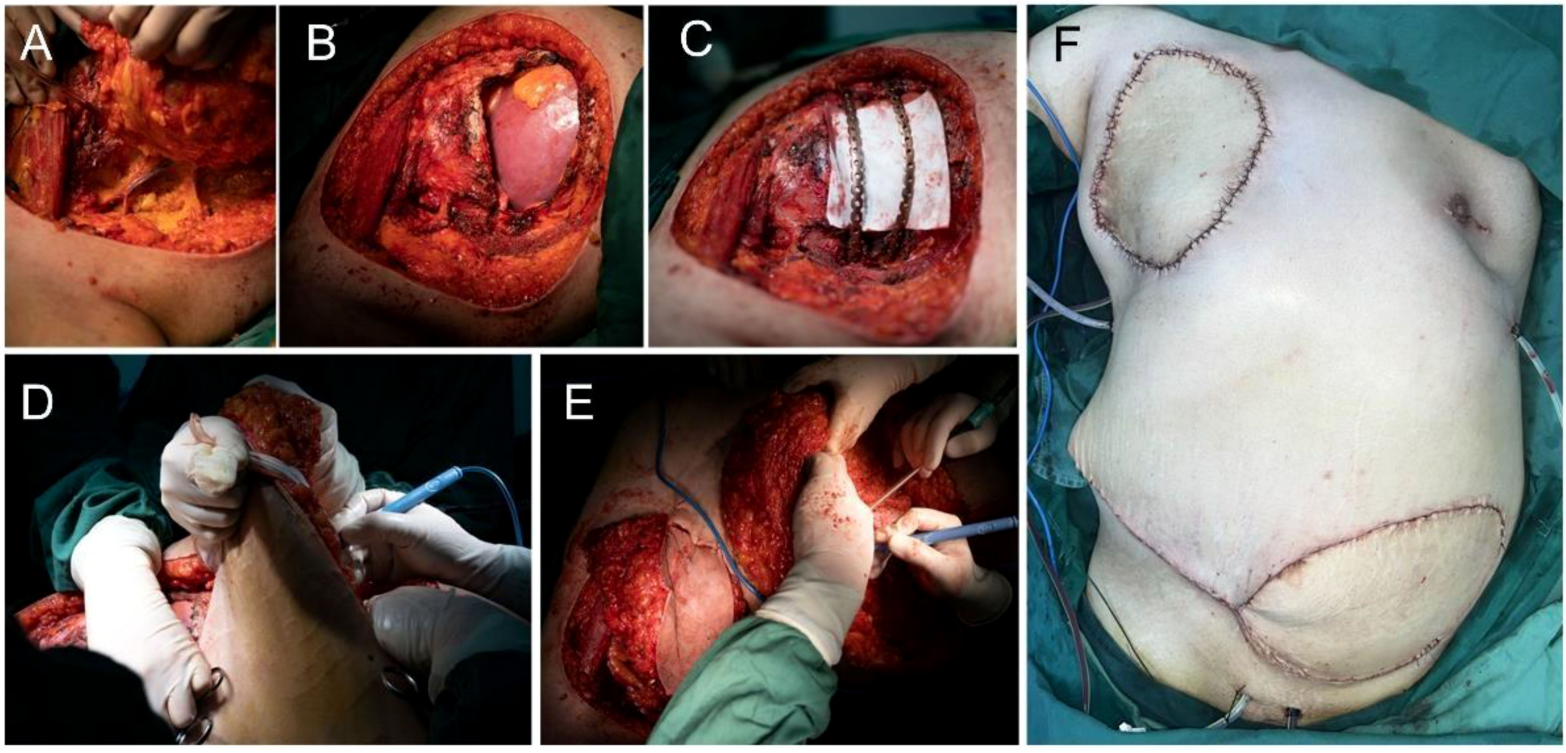
Surgical procedure. **(A-C)** Extended radical mastectomy and chest wall reconstruction. **(D, E)** The right pedicled rectus abdominis flap **(F)**.

**Figure 5:**
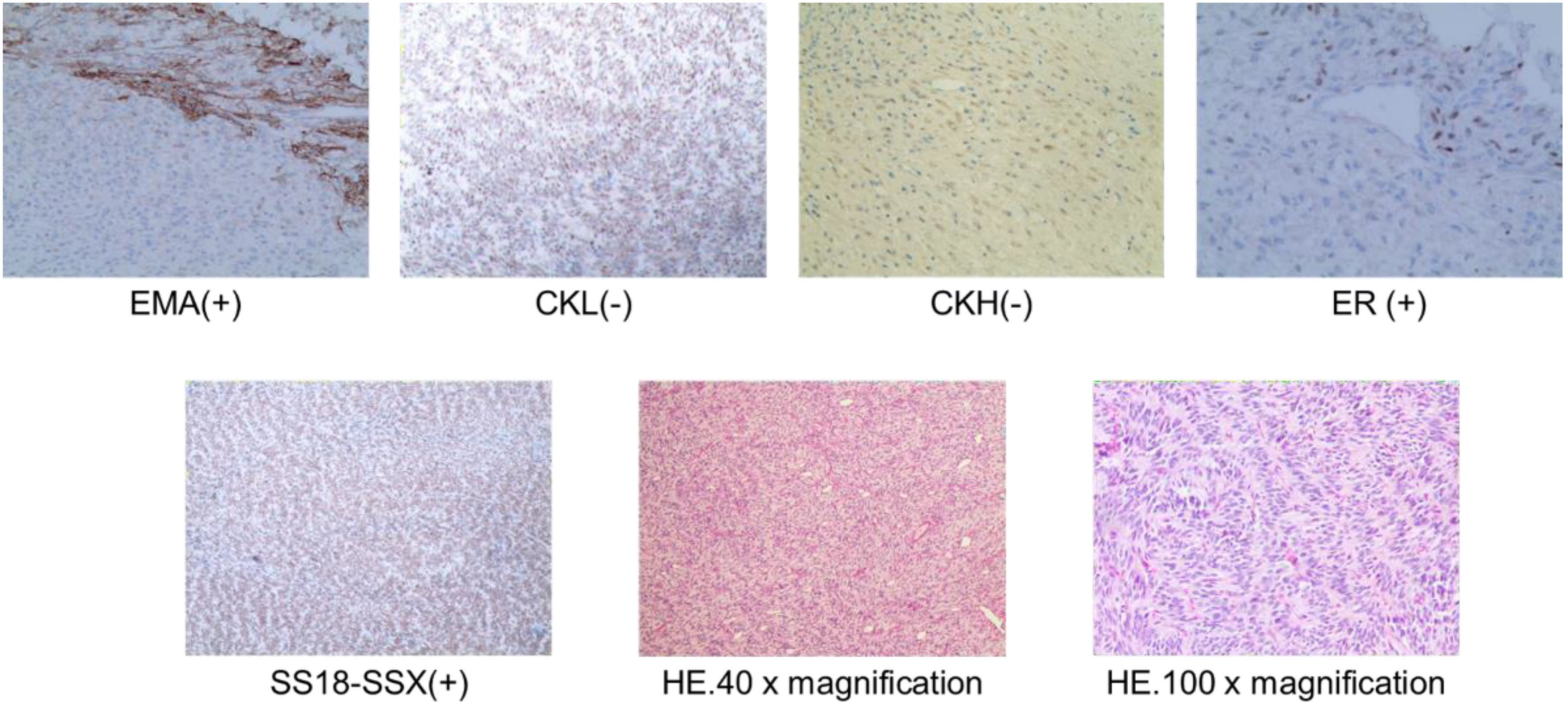
Postoperative pathological HE staining and immunohistochemistry of right breast carcinoma (Pathological ID: 23003336).

**Figure 6:**
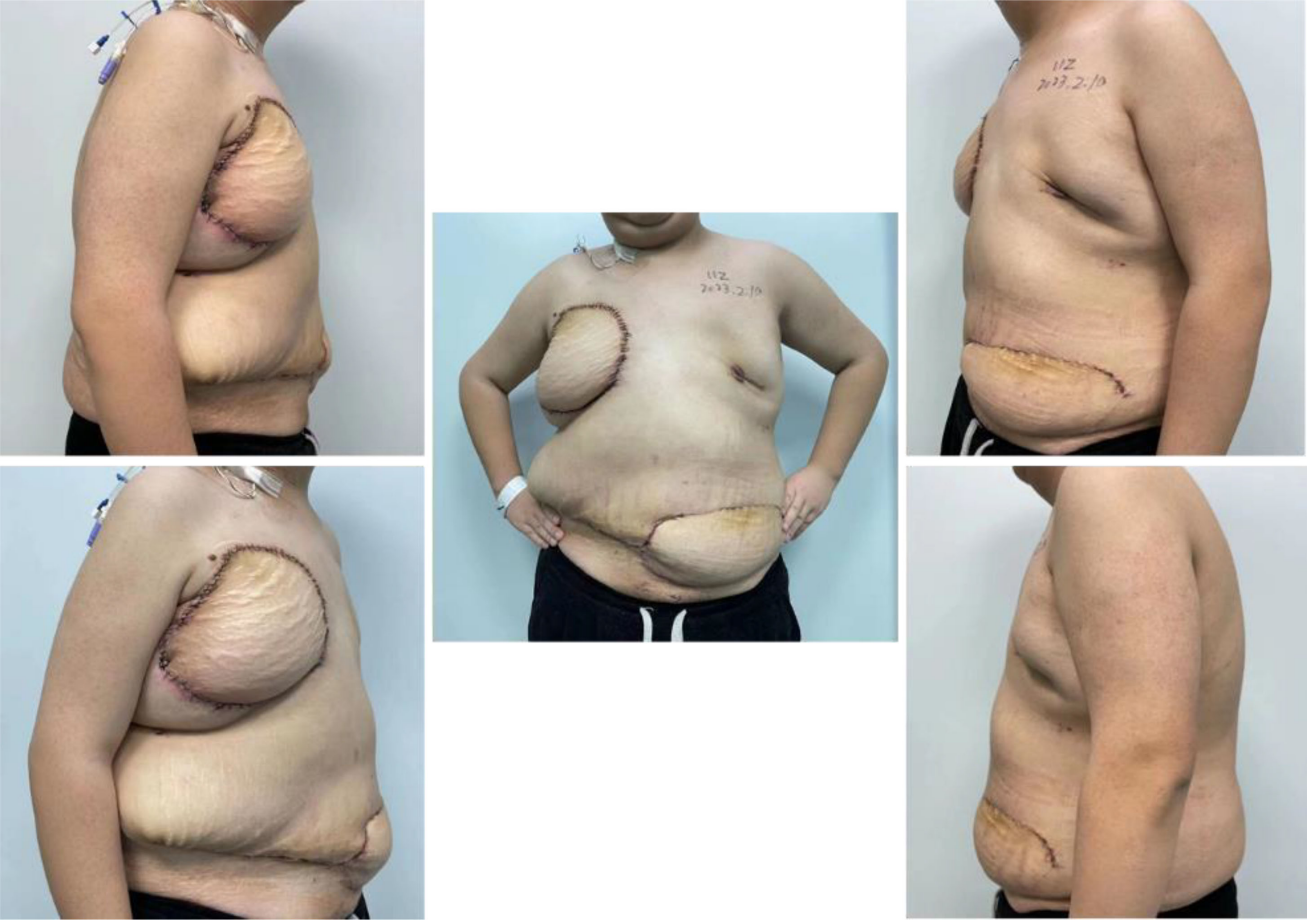
Postoperative follow-up photos at 12 days.

The patient underwent subcutaneous mastectomy of the left breast to address gynecomastia. For the right breast, an extended radical mastectomy was performed on right breast after intraoperative freezing pathology confirmed the absence of sentinel lymph node metastasis (0/2) in the right axilla (**Figure 3**). The target lesion involved the proximal portions of the 4th and 5th ribs, and therefore, the 4th and 5th ribs and parietal pleura between them were removed. The subcutaneous tissues at 4, 3, 1, 12, 6, 10, 9, 7 o ‘clock of the right breast tumor resection, as well as the tissues at the lower, upper, and internal and external margins of the ribs, were sent for frozen pathological examination to ensure that the surgical margins were negative. The Matrix RIB fixation system was used for thoracoplasty (**Figures 4A-C**). The right pedicled rectus abdominis flap was used for chest wall reconstruction (**Figures 4D, E**). The inferior epigastric vessels of the flap were anastomosed to the lateral thoracic vessels to ensure an adequate blood supply. Postoperative pathological immunohistochemistry confirmed that the malignant tumor originated from the breast, and the final diagnosis was right emulsified carcinoma (carcinosarcoma). The main tumor was biphasic synovial sarcoma. As shown in **Figure 5**, immunohistochemical results of Radical resection of right breast carcinoma: (Pathological ID: 23003336): SS18-SSX (+), STAT6(-), S100 (-), SMA (-), TLE1 (+), Desmin (-), CK5/6 (focal +), CK20 (-), CK (focal +), CK8/18 (-), CKH(-), CKL (-), ER (focal +), Mammaglobin (focal +), GATA3(-), GCDFP-15(-), EMA (focal +), Ki-67(+, 70%), P63(Individual cell+), and Vim (+). No lymph node metastasis was observed in the nine sentinel lymph nodes of the right axilla (0/9). The survival status of the flaps was monitored by assessing surface color and blood supply every 2 hours within 48 hours and every 4 hours on the third day. Postoperative recovery was uneventful with good flap vitality (**Figure 6**).

## 4 Discussion

Synovial sarcoma is a highly aggressive soft-tissue malignancy primarily affecting adolescents and young adults, often found in the lower extremities (1, 2). Recent literature describes a case of a 44-year-old woman with SS in the thigh that recurred and metastasized to the breast and lungs following surgery (4). Primary breast SS is exceedingly rare, particularly in adolescent males (5). Male breast cancer accounts for only 1% of all breast cancer cases. To the best of the authors’ knowledge, only three cases of female breast SS have been reported (6–8), and we report the first documented case of breast SS in males.

SYT-SSX is a reliable indicator for the pathological diagnosis of SS (9, 10). In this case, SS in this young boy was confirmed through preoperative biopsy and postoperative pathology, which identified SS18 (18q11) (SYT) and SS18 gene translocation (11, 12). Surgery remains the best treatment option for SS. The role of chemotherapy in the treatment of SS remains debatable; it is generally used for salvage therapy in advanced stages of the disease (13). Although synovial sarcomas are equally insensitive to radiotherapy, experts consider a combination of radiotherapy and surgery for high-risk patients (e.g., grade 3 tumors, deep tumors, or tumors larger than 5 cm). Although the surgical margin of the patient reported in this case was negative, the patient had a large tumor, and postoperative radiotherapy is still strongly recommended to reduce the risk of recurrence. As the patient was lost to follow-up, whether if they received radiotherapy was subsequently administered could not be determined.

Previous research has demonstrated that tumor size (P<0.005) and age (P=0.024) are significantly negatively associated with cancer-specific survival (CSS) in SS, regardless of tumor location and site (14). The lesions in the right breast mass in this case were large and extended into the 4th and 5th ribs. Restoring both functionality and aesthetics was critical. Along with thoracic reconstruction, a pedicled transverse rectus abdominis myocutaneous (TRAM) flap was used to fill the cavity created by the removal of the pectoralis major and minor muscles. The TRAM flap is a common choice for breast construction (15). Importantly, in this case, the inferior epigastric vessels of the flap were supercharged by anastomosing them to the lateral thoracic vessels when signs of venous congestion appeared. This strategy has not been previously reported in cases of SS. This is the first reported case of breast and chest wall reconstruction for SS. When the patient returned to the hospital 12 days after surgery, the incision healed well and the appearance was satisfactory (**Figure 6**). The patient was lost to follow-up due to electing to continue subsequent treatment at another institution.

Synovial sarcoma is a highly aggressive cancer with a poor prognosis. The 5-year overall survival for SS is approximately 60.5%, and half of the patients will develop metastatic recurrence, with a 5-year survival rate of 14.4% (16, 17). Therefore, there is an urgent need to develop more effective strategies for SS. The disease is driven by the pathognomonic t (X;18) chromosomal translocation and subsequent formation of the SS18:SSX fusion oncogenes (18). A recent phase 1 clinical trial (NCT03132922) has highlighted the efficacy of T-cell receptor therapy in treating SS (19). Given the role of SS18-SSX in epigenetic regulation and the effect of BET inhibitors on cell cycle regulators such as MYC, p21, CDK4, and CDK6, BET inhibitors targeting the intrinsic apoptosis pathway regulated by SS18-SSX represent a promising potential therapeutic option (20). In addition, inhibiting CDK9 in sarcomas has been shown to downregulate the expression of some oncogenes and reduce the proliferation and growth of various sarcoma cells. Targeting CDK9 in cancer has yielded promising results in both preclinical and clinical studies (21). We look forward to further advancements in effective treatments for SS that could improve prognosis and prolong survival time.

## Data Availability

The original contributions presented in the study are included in the article/supplementary material. Further inquiries can be directed to the corresponding author/s.
